# Rotor Fault Diagnosis Based on Characteristic Frequency Band Energy Entropy and Support Vector Machine

**DOI:** 10.3390/e20120932

**Published:** 2018-12-05

**Authors:** Bin Pang, Guiji Tang, Chong Zhou, Tian Tian

**Affiliations:** Department of Mechanical Engineering, North China Electric Power University, Baoding 071000, China

**Keywords:** improved singular spectrum decomposition, characteristic frequency band energy entropy, support vector machine, rotor, fault diagnosis

## Abstract

Rotor is a widely used and easily defected mechanical component. Thus, it is significant to develop effective techniques for rotor fault diagnosis. Fault signature extraction and state classification of the extracted signatures are two key steps for diagnosing rotor faults. To complete the accurate recognition of rotor states, a novel evaluation index named characteristic frequency band energy entropy (CFBEE) was proposed to extract the defective features of rotors, and support vector machine (SVM) was employed to automatically identify the rotor fault types. Specifically, the raw vibration signal of rotor was first analyzed by a joint time–frequency method based on improved singular spectrum decomposition (ISSD) and Hilbert transform (HT) to derive its time–frequency spectrum (TFS), which is named ISSD-HT TFS in this paper. Then, the CFBEE of the ISSD-HT TFS was calculated as the fault feature vector. Finally, SVM was used to complete the automatic identification of rotor faults. Simulated processing results indicate that ISSD improves the end effects of singular spectrum decomposition (SSD) and is superior to empirical mode decomposition (EMD) in extracting the sub-components of rotor vibration signal. The ISSD-HT TFS can more accurately reflect the time–frequency information compared to the EMD-HT TFS. Experimental verification demonstrates that the proposed method can accurately identify rotor defect types and outperform some other methods.

## 1. Introduction

Rotor is a key element of rotary machine and its working condition affects the reliability and safety of whole mechanical system. Accurate identification of rotor faults is very helpful for reducing economic losses and ensuring production safety [1,2,3,4].

As the vibration signal of rotors carries plentiful fault information, a lot of rotor fault diagnosis approaches were developed based on vibration signal analysis [5,6]. Due to the non-stationary and non-linear characteristics of rotor vibration signals, classical signal analysis methods based on Fourier transform fail to extract the fault features of rotors accurately [7]. In recent years, the application of time–frequency analysis to rotor failure detection has attracted considerable attention as the time–frequency analysis approaches are able to reflect the characteristic information in both time domain and frequency domain [8]. Among these developed time–frequency analysis approaches, wavelet transform (WT) is one of the most effective one for its high time–frequency resolution [9,10]. However, an appropriate basis function should be selected or designed in every use of WT to ensure a satisfactory result, which reduces the adaptability of WT [11]. Considering the drawback of WT, Huang et al. developed a novel adaptive time–frequency technique called Hilbert-Huang transform (HHT) [12], which is the combination of empirical mode decomposition (EMD) and Hilbert transform (HT). EMD is used to adaptively decompose a multi-component signal into several intrinsic mode functions (IMFs) that carry the useful information of different frequency bands. Then the instantaneous amplitude (IA) and instantaneous frequency (IF) of each IMF are calculated by HT. Consequently, the HHT time–frequency spectrum (TFS) can be obtained by integrating the IAs and IFs of all IMFs. HHT has an extensive application in detecting mechanical faults owing to its priorities of extracting non-stationary characteristics of signals.

The HHT TFS demonstrates the energy–frequency–time distribution of a signal. For different mechanical faults, the corresponding energy–frequency–time distribution varies greatly. To implement the automatic identification of different mechanical fault types, it is of great significance to exploit an effective index to measure the differences of the corresponding energy–frequency–time distribution. Shannon Entropy is a satisfactory indicator for measuring the complexity and regularity of a signal and the diversity of any distribution [13,14,15]. Therefore, some entropy-based indicators were developed to evaluate the fault features of rotating machinery. For example, the Teager energy entropy ration (TEER) was proposed as an impulsive feature evaluation index to select the optimal sub-band component obtained by performing wavelet transform decomposition on the vibration signal of rolling bearings in [16]. The TEER index is the combination of the Teager energy entropy in time domain and frequency domain. However, it cannot reflect the information of the time–frequency domain. Yu et al. proposed a time–frequency entropy (TFE) method by combing the merits of entropy and HHT for gear fault diagnosis [17]. The TFE gives an indicator that can estimate the information of time–frequency domain. However, the TFE has two main shortcomings. One is originated from EMD. EMD is troubled by mode mixing [18], which makes the obtained HHT TFS cannot accurately reflect the time–frequency information of a signal. On the other hand, the TFE index is a measure of the whole information of the HHT TFS, it is not able to extract the local energy–frequency–time distribution of the TFS, which leads the calculation of TFE to be relatively complex. In conclusion, the TFE can be improved from two aspects: (a) Improve the HHT method to obtain a more accurate TFS; (b) Develop a novel index which can reflect the local characteristics of the TFS.

To improve the accuracy of HHT, some other advanced signal decomposition approaches, such as ensemble empirical mode decomposition (EEMD) [19], complementary ensemble empirical mode decomposition (CEEMD) [20], empirical wavelet transform [21], variable mode decomposition (VMD) [22], and adaptive local iterative filtering (ALIF) [23] have been employed to replace EMD to extract the sub-components of a signal. Despite these methods have a better performance over EMD in data decomposition, the premise of obtaining ideal results by using these methods is the good choice of parameters, the process of which is very complex. Singular spectrum decomposition (SSD) is a new adaptive data decomposition method [24], the basic principle of which is based on singular spectrum analysis (SSA). It could choose the parameters automatically and retrieve the construction components accurately. Moreover, the existence of non-meaningful components could be minimized to a large extend. However, the problem of end effects still has not been solved in the original SSD algorithm, which needs to be studied further. Some useful techniques such as extreme value point extension [25], data prediction [26] and waveform matching extension method [27] have been studied by scholars for restraining the end effect of EMD. However, these developed approaches have some drawbacks. For example, the extreme value point extension method only considers the information of several extreme value points near signal endpoint but does not take into account the internal law of the signal, so the effect of this method for complex non-stationary signals is not ideal. The data prediction method is on the base of the intelligent prediction techniques such as neural work [28] and support vector regression [29]. Its improvement effect depends largely on the parameter setting of the prediction tool itself, and the operation time is also long, which decreases the practicability of this method. The waveform matching extension method takes into account the internal laws and trends of signal and has high computation efficiency. However, how to choose the best matching waveform adaptively is a difficult problem. In view of the above problems, a novel adaptive waveform matching extension approach was introduced for solving the end effects and improving the decomposition accuracy of SSD. The improved SSD (ISSD) method was employed to replace EMD to extract the sub-component signals of the rotor vibration signal. Several sub-component signals called singular spectrum components (SSCs) can be extracted after the ISSD decomposition. Then an ISSD-HT TFS can be produced by using HT to demodulate every SSC. The ISSD-HT TFS is more accurate than the HHT TFS due to the advantages of ISSD over EMD. Considering that the main fault features of rotors locate in the former harmonics of rotary frequency of the TFS, a new index called characteristic frequency band energy entropy (CFBEE) was proposed in this paper to extract the local characteristics of the ISSD-HT TFS corresponding to different working conditions of rotors. After extracting the CFBEE as the feature vector, an outstanding machine-learning classifier called support vector machine (SVM) [30], which is especially suitable for the classification problem of small samples was used in this paper to achieve the fault identification of rotors.

This organization of this paper is as follows: In Section 2, basic theory background of ISSD, characteristic frequency band energy entropy and SVM is introduced. Section 3 describes the framework of the proposed method. Simulation and experiment signals are analyzed in Section 4 and Section 5, respectively. Besides, comparison is performed among the proposed method and three other methods to demonstrate the effectiveness and superiority of the proposed method. Conclusion is summarized in Section 6.

## 2. Theory Background

### 2.1. Singular Spectrum Decomposition

SSD is a novel signal decomposition approach derived from SSA. While SSA has a powerful capability of recovering sub-component signal from the multi-component signals [31], how to properly choose the embedding dimension and select the principal components for reconstructing a specific sub-component are its two main obstacles. The use of SSD improves the process by allowing the SSA fundamental parameters to be selected adaptively. Given an original multi-component signal *y*(*n*), *n* = 1,2,…,*N*, its sub-component signals, referred to as SSCs, can be iteratively extracted using the following steps:

(**a**) Embedding.

If the embedding dimension is selected as *K*, 1 < *K* < *N*, a *K* × *L* matrix ***Y*** named Hankel matrix can be generated. The Hankel matrix consists of *K* lagged vectors ***y****_i_* = [*y*(*i*),…,*y*(*N*), *y*(1),…,*y*(*i*−1)], *i* = 1, 2,…,*N* − *K* + 1. Taking the one-dimensional signal *y*(*n*) = {1, 2, 3, 4, 5} as an example, if the embedding dimension *K* is set to 3, the corresponding Hankel matrix becomes:

(1) $$Y=\left[\begin{array}{ccccc} 1 & 2 & 3 & 4 & 5\\ 2 & 3 & 4 & 5 & 1\\ 3 & 4 & 5 & 1 & 2 \end{array}\right].$$

In the SSD method, the embedding dimension is adaptively selected as *K* = *f_s_*/*f_max_*, where *f*_max_ represents the dominant frequency in the power spectral density (PSD) of *y*(*n*) and *f_s_* indicates the sampling frequency.

(**b**) Decomposition.

The Hankel matrix ***Y*** is then decomposed using singular value decomposition (SVD):

(2) $$\begin{array}{l} Y=UDV^{T}\\ =\left[u_{1},u_{2},\cdots ,u_{K}\right]\left[\begin{array}{ccccc} \lambda_{1} & 0 & 0 & 0 & 0\\ 0 & \lambda_{2} & \cdots & 0 & 0\\ \vdots & \vdots & \ddots & \vdots & 0\\ 0 & 0 & \cdots & \lambda_{K} & 0 \end{array}\right]\left[\begin{array}{c} v_{1}{}^{T}\\ v_{2}{}^{T}\\ \vdots \\ v_{N}{}^{T} \end{array}\right]\\ =\lambda_{1}u_{1}v_{1}{}^{T}+\lambda_{2}u_{2}v_{2}{}^{T}+\ldots +\lambda_{K}u_{K}v_{K}{}^{T}\\ =H_{1}+H_{2}+\cdots +H_{K} \end{array}$$

where ***U*** ∈ ***R***^*K* × *K*^, ***D*** ∈ ***R***^*L* × *L*^ and ***V*** ∈ ***R***^*N* × *N*^. Both ***U*** and ***V*** are orthogonal matrices, which contains the left and right vectors, respectively. The main diagonal of ***D*** carries the singular values.

After the SVD process, the Hankel matrix ***Y*** is decomposed into *K* principle components.

(**c**) Grouping.

The *R* (*R* < *K*) principle components, whose left eigenvectors reflect a domain frequency in the range [*f*_max_ − Δ*f*, *f*_max_ + Δ*f*], are selected out to reconstruct the sub-component signal. Δ*f* is determined by the Gaussian interpolation of the PSD of *y*(*n*).

(**d**) Reconstruction.

The selected *R* principal components are employed to generate a rank-R approximation of ***Y***. Then the corresponding sub-component can be recovered by applying diagonal averaging to the adjusted version of this matrix. For instance, the adjusted version of the above matrix shown in Equation (1) can be described as:

(3) $$X=\left[\begin{array}{ccccc} & & 1 & & \\ & 1 & 2 & & \\ 1 & 2 & 3 & 4 & 5\\ 2 & 3 & 4 & 5 & \\ 3 & 4 & 5 & & \end{array}\right].$$

The detailed introduction to SSD can refer to [24].

### 2.2. Improved Singular Spectrum Decomposition

As reported in [24], a limitation of SSD is that the decomposed components are always affected by end effects. This requires the extension of both ends of the original signal to isolate the distortion as far as possible from the outside of the signal to be analyzed. However, this extension cannot be blind extension, but to make the extended waveform conform to the natural trend of the original signal as much as possible, to maximize the maintenance of the change trend of the original signal and achieve a smooth transition between the extended waveform and the original signal. Thus, a novel waveform matching degree evaluation index was proposed by combing the correlation coefficient and root mean square error to adaptively perform the waveform matching extension. An improved singular spectrum decomposition (ISSD) approach was developed by using this waveform matching extension method to improve the decomposition accuracy.

The extension of the signal includes the left and right ends, and the following is an example of the extension of the left side to illustrate the proposed adaptive waveform matching extension method.

**Figure 1:**
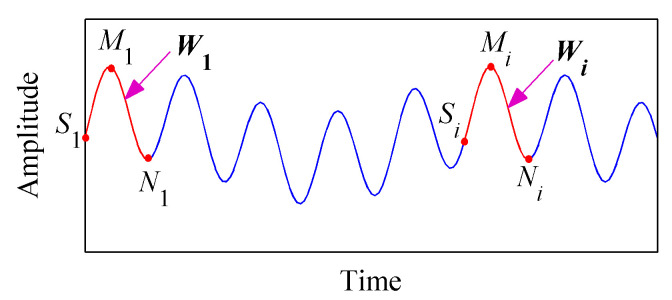
Schematic diagram of waveform matching.

Assume the original signal is *x*(*t*), *M_i_* and *N_i_* (*i* = 1, 2, 3,…) are the maximum and minimum points of *x*(*t*), respectively. $t_{M_{i}}$ and $t_{N_{i}}$ correspond to the time of the maximum and minimum points, respectively. The left end of the signal is defined as *S*_1_. The signal shown in Figure 1 was taken as an example, the first extreme point of which is a maximum point. The waveform *S*_1_-*M*_1_-*N*_1_ is targeted as the characteristic waveform ***W***_1_. Then the optimal matching waveform will be searched within the whole data. Finally, the left data of the optimal matching waveform will be extended to the left of the data. The specific steps are given as follows:

(1) Find out the starting point of all matched waveform *S_i_* (*i* = 1,2,3…), whose corresponding time can be calculated as:

(4) $$t_{S_{i}}=\frac{t_{M_{1}}t_{N_{i}}-t_{N_{1}}t_{M_{i}}}{t_{M_{1}}-t_{N_{1}}}.$$

The waveform *S_i_*-*M_i_*-*N_i_* is defined as the matched waveform ***W**_i_*.

(2) Calculate the proposed matching degree of the characteristic waveform and the matching waveform by using the following equation:

(5) $$D_{i}=\frac{corr(W_{1},W_{i})}{RMSE(W_{1},W_{i})},$$

where, $corr(W_{1},W_{i})$ and $RMSE(W_{1},W_{i})$ represent the cross-correlation coefficient and root mean square error between ***W***_1_ and ***W**_i_*, respectively. If the correlation between the two waveforms is stronger and the root mean square error is smaller, the matching degree is higher.

(3) Treat the matching waveform which has the largest value of *D_i_* as the optimal matching waveform. When there are multiple optimal matching waveforms, the one which is farthest from the starting point is regarded as the optimal matching waveform.

(4) Extend the data on the left side of the optimal matching waveform to the left side of *S*_1_.

Similarly, the same steps can be used to complete the right extension of the data. The extended data will be decomposed by SSD to generate a series of SSCs with physical meanings. Finally, the SSCs with the same length as the original signal data can be obtained by intercepting based on the pre-extension position. As the ISSD will be combined with HT to conduct the time–frequency analysis for the rotor fault signal in this paper and HT also has end effects, the interception process will take place after the HT process.

### 2.3. Time–frequency Analysis Bsed on Improved Singular Spectrum Decomposition and Hilbert Transform

ISSD was combined with HT to perform the time–frequency on rotor vibration signal in this work. The process of this time–frequency analysis is as follows. Firstly, the original signal is decomposed into a series of SSCs by ISSD. Then, the obtained SSCs are demodulated by HT to derive their instantaneous frequency (IF) and IA.

For a given singular spectrum component *SSC*(*t*), its HT is defined as follows [32]:

(6) $$H[SSC(t)]=\frac{1}{\pi }\int_{-\infty }^{+\infty }\frac{SSC(t)}{t-\tau }d\tau .$$

We can further get the analytical signal:

(7) $$z(t)=SSC(t)+jH[SSC(t)]=a(t)e^{j\varphi (t)},$$

where, *a*(*t*) represents the IA of *SSC*(*t*) and *φ*(*t*) is the instantaneous phase. Their specific expressions are given in Equations (8) and (9), respectively:

(8) $$a(t)=\sqrt{SSC^{2}(t)+H^{2}[SSC(t)]}.$$

(9) $$\varphi (t)=\arctan\frac{H[SSC(t)]}{SSC(t)}.$$

The IF of *SSC*(*t*) can be derived as:

(10) $$\omega (t)=\frac{d\varphi (t)}{dt}.$$

The IA and IF reflects the time–frequency information of *SSC*(*t*). By integrating the time–frequency information all SSCs, the ISSD-HT TFS can be derived.

### 2.4. Characteristic Frequency Band Energy Entropy

The time–frequency analysis can be an effective tool for extracting the fault signatures of rotors. However, to classify the rotor fault types automatically, some indexes must be developed to evaluate the useful information carried by the TFS. Time–frequency entropy (TFE) was thus introduced by Yu et al. [17] to measure the energy distribution characteristics of the HTT TFS to distinguish different gear fault types. To highlight the innovation of the proposed method, the principle of the TFE would be given briefly before introducing the proposed CFBEE in this work.

In the principle of the TFE method, the whole TFS plane is divided into *N* small time–frequency blocks with the same area, and the normalized energy of each block *Q_i_* can be calculated as:

(11) $$Q_{i}=W_{i}/A,$$

where, *W_i_* and *A* represent the energy of the time–frequency block and the energy of the whole TFS plane, respectively.

Equation (12) gives the definition of TFE based on HHT:

(12) $$TFE(Q)=-\sum_{i=1}^{N}Q_{i}\ln Q_{i}.$$

From the definition of TFE, we can deduce that it is a measure of the whole TFS. Therefore, some redundant information may be wrongly considered in the TFE index when the rotor vibration signal is affected by noise and other abnormal disturbances. Previous studies demonstrated that some sub-harmonics and sup-harmonics of the rotational speed, which is called the fault characteristic frequencies in this paper, would be generated when rotor defects occur. Hence, it is very critical to takes some measures to extract the fault features around the sub-harmonics and sup-harmonics of the rotational speed. Considering the characteristic information of rotor faults is always concentrated in the former fault characteristic frequencies, we proposed the CFBEE index, which is used to mainly reflect the local characteristic information around the eight former characteristic frequencies, i.e., the 0.5X, 1X, 1.5X, 2X, 2.5X, 3X, 3.5X, 4X of the rotational frequency. Firstly, the time–frequency spectrum plane is divided into *M* × *N* blocks. Then, eight characteristic frequency bands of [0.1X–0.9X], [0.6X–1.4X], [1.1X–1.9X], [1.6X–2.4X], [2.1X–2.9X], [2.6X–3.4X], [3.1X–3.9X], [3.6X–4.4X], which respectively take the eight former characteristic frequencies as the center frequency are defined as characteristic frequency bands. Finally, the energy entropy of the eight characteristic frequency bands is calculated as the characteristic frequency band energy entropy.

**Figure 2:**
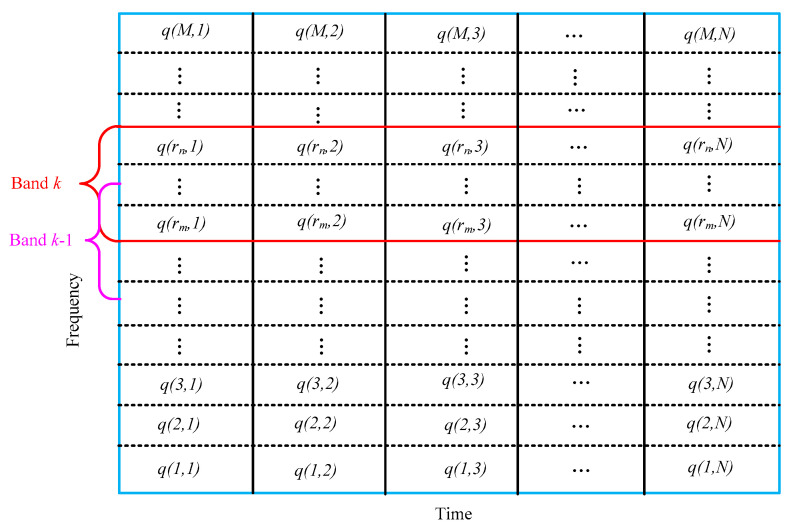
Time–frequency spectrum plane division corresponding to the characteristic frequency band energy entropy method.

Figure 2 shows the division result of the TFS plane corresponding to the CFBEE method. For the *k*th characteristic frequency band (k=1,2,3,4,5,6,7,8), $r_{m}$ and $r_{n}$ represent its start and end row. Supposing the energy of the time–frequency block in the *k*th characteristic frequency band is *W*(*i*,*j*) and *W_k_* is the total energy of the *k*th characteristic frequency band. The normalized energy of any time–frequency block can be described as follows:

(13) $$q(i,j)=W(i,j)/W_{k}.$$

Then, the total energy of the *i*th row in the *k*th characteristic frequency band can be derived as:

(14) $$Q_{i}=\sum_{j=1}^{N}q(i,j).$$

The CFBEE of *k*th characteristic frequency band is defined as:

(15) $$CFBEE_{k}=-\sum_{i=r_{m}}^{r_{n}}Q_{i}\ln Q_{i}.$$

After calculating the CFBEE of all the eight characteristic frequency bands, a fault feature vector, i.e., {*CFBEE*_1_, *CFBEE*_2_, *CFBEE*_3_, *CFBEE*_4_, *CFBEE*_5_, *CFBEE*_6_, *CFBEE*_7_, *CFBEE*_8_}, is obtained to characterize the fault types. From the principle of CFBEE, we can find that it is a direct measure of the local information around the fault characteristic frequency. Previous studies indicate that the background noise can generate high-frequency components in the time–frequency spectrum. Hence, the CFBEE index can exclude some redundant information corresponding to the high-frequency noise.

### 2.5. Support Vcetor Machine

SVM is a machine-learning algorithm based on the structural risk minimization principle of statistical learning theory [33]. For the two-class problem, SVM maps the non-linear separable problem in low-dimensional space to high-dimensional space through a certain non-linear kernel function mapping, making it linear separable. The core of SVM is to seek a hyper plane in high-dimensional space as the segmentation of two classifications to ensure the minimum classification error rate.

For a given training sample $\left\{\left(x_{i},y_{i}\right)\right\},\left(i=1,2,3,\ldots ,n\right),x_{i}\in C^{d},y_{i}\in \left\{-1,1\right\}$, where *d* represents the dimension of the space, *y_i_* is the pattern label of *x_i_*. The hyper plane can be described as:

(16) ω•x+k=0,

where, *ω* and *k* represent the weighting vector and the bias, respectively.

The optimal classification plane in the feature space can be constructed by solving the following quadratic programming problem:

(17) $$\left\{ \begin{array}{l} \min L(\omega )=\frac{1}{2}{\left\|\omega \right\|}^{2}+C\sum_{i=1}^{n}\xi_{i}\\ s.t.\;y_{i}•(\omega x+k)\geq 1-\xi_{i},i=1,2,\ldots ,n \end{array}\right.,$$

where, *C* is a penalty factor which can indicates the penalty level for classification errors and $\xi_{i}$ represents a slack factor. According to quadratic programming, Equation (16) is equivalent to solve the following optimization problem:

(18) $$\left\{ \begin{array}{l} \max\;W(\lambda )=\sum_{i=1}^{n}\lambda_{i}-\frac{1}{2}\sum_{i,j=1}^{n}\lambda_{i}\lambda_{j}y_{i}y_{j}K(x_{i},x_{j})\\ s.t.\;\sum_{i=1}^{n}\lambda_{i}y_{i}=0,0\leq \lambda_{i}\leq C,i=1,2,\ldots ,n \end{array}\right.,$$

where *λ_i_* is Lagrange multiplier and *K*(*x_i_*, *x_j_*) represents the kernel function. The optimal classification decision function can be derived as:

(19) $$f(x)=\mathrm{sgn}\left(\sum_{i=1}^{n}\lambda_{i}y_{i}K(x_{i},x)+k\right).$$

To deal with multi-class fault diagnosis problems, ‘one-against-others’ SVM algorithm illustrated in [30] is adopted in this paper.

## 3. The Framework of the Proposed Method

**Figure 3:**
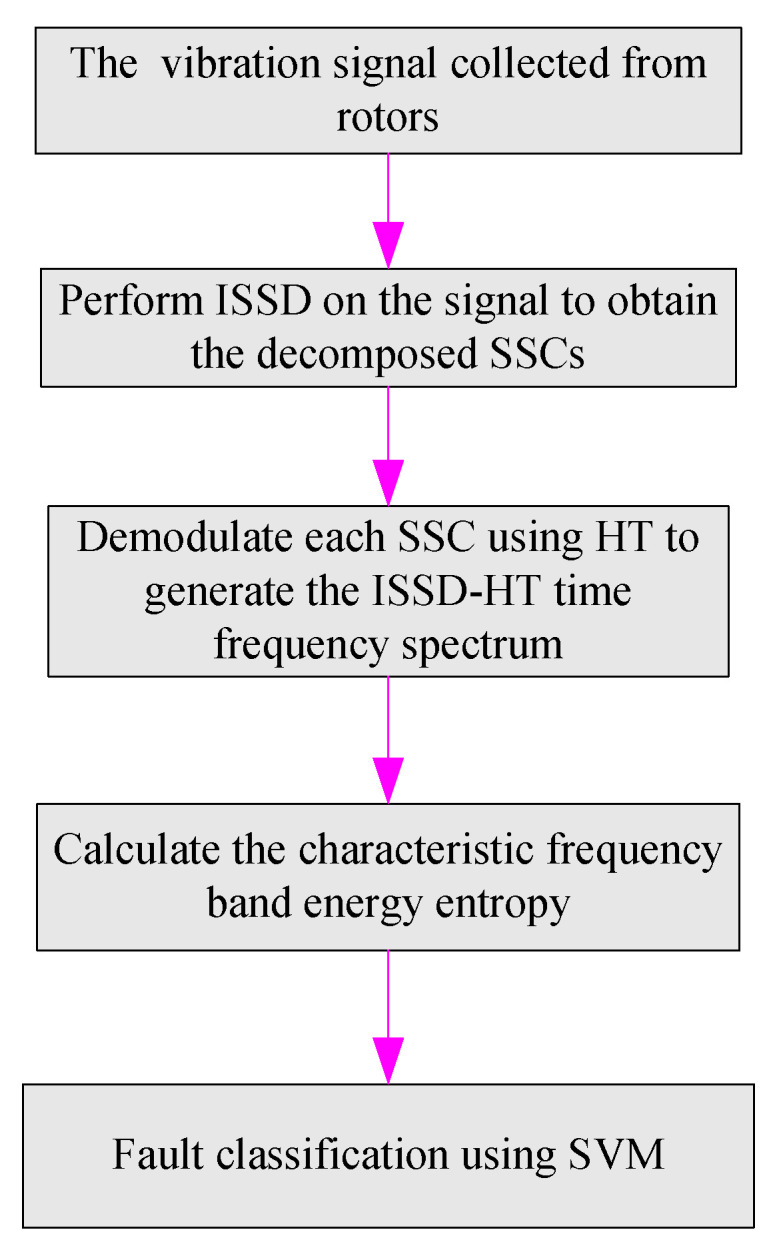
The framework of the proposed method.

Figure 3 displays the framework of the proposed method, which can be introduced in detail as:

(1) Decompose the signal collected by the sensors mounted on the rotor by the ISSD method to obtain the decomposed SSCs.

(2) Perform Hilbert transform on the SSCs to derive the ISSD-HT TFS plane.

(3) Divide the ISSD-HT TFS plane into eight frequency bands, namely, [0.1X–0.9X], [0.6X–1.4X], [1.1X–1.9X], [1.6X–2.4X], [2.1X–2.9X], [2.6X–3.4X], [3.1X–3.9X], [3.6X–4.4X], and the characteristic frequency band energy entropy of each band is calculated with the method mentioned above.

(4) Identify the rotor fault types with the SVM classification method.

## 4. Simulation Signal Analysis

**Figure 4:**
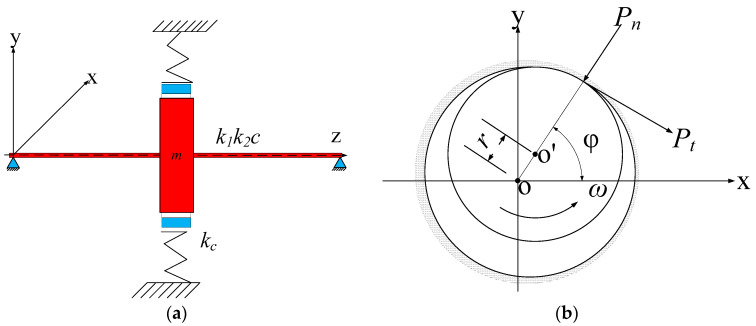
(**a**) Jeffcott rotor model; (**b**) rubbing force model.

To validate the superiority of ISSD in processing rotor fault signals, a Jeffcott rotor rub-impact model was established to construct simulation signal firstly. Then the signal is analyzed by EMD, SSD and ISSD respectively for comparison. The rotor rubbing model is shown in Figure 4a. A single disc rotor, the mass of which is *m*, is fixed in the middle of a simply supported shaft with the angular speed of *ω*. The mass of the shaft is neglected. It is assumed that the linear bending stiffness of the rotating shaft is *k*_1_ and the non-linear bending stiffness of the rotating shaft is *k*_2_. *c* and *k*_c_ are the damping coefficient and the radial stiffness of the stator, respectively. Figure 4b shows a schematic diagram of the rotor rubbing force. Assuming that the rubbing force between the rotor and the stator conforms to Coulomb’s law and $r=\sqrt{x^{2}+y^{2}}$ is the radial displacement of the geometric center of the rotor, the normal contact force *P_n_* and tangential rubbing forces *P_t_* can be calculated as follows [34]:

(20) $$\left\{ \begin{array}{l} P_{n}=(r-\delta )k_{c}\\ P_{t}=(f+\alpha v)P_{n} \end{array}\right.,$$

where, *δ* is the minimum gap between rotor and stator, $v={\left({\dot{x}}^{2}+{\dot{y}}^{2}\right)}^{1/2}$ is the relative sliding speed between the moving and stationary parts, *f* is the friction coefficient and *α* is the speed influence factor. If the angle between the point of occurrence of rubbing and the *x*-axis is *φ*, the normal and tangential rubbing forces can be expressed in the X-Y coordinate system as follows:

(21) $$\left\{ \begin{array}{l} P_{x}\\ P_{y} \end{array}\right.=\left[\begin{array}{cc} -\cos\phi & \sin\phi \\ -\sin\phi & -\cos\phi \end{array}\right]\left\{\begin{array}{l} P_{n}\\ P_{t} \end{array}\right\}=-\frac{(r-\delta )k_{c}}{r}\left[\begin{array}{cc} 1 & -(f+\alpha v)\\ f+\alpha v & 1 \end{array}\right]\left\{\begin{array}{c} x\\ y \end{array}\right\}.$$

Then, the equation of motion of the rotor can be established as Equation (22) shows:

(22) $$\left[\begin{array}{cc} m & \\ & m \end{array}\right]\left\{\begin{array}{l} \ddot{x}\\ \ddot{y} \end{array}\right\}+\left[\begin{array}{cc} c & \\ & c \end{array}\right]\left\{\begin{array}{l} \dot{x}\\ \dot{y} \end{array}\right\}+\left[\begin{array}{cc} k_{1} & \\ & k_{1} \end{array}\right]\left\{\begin{array}{l} x\\ y \end{array}\right\}+\left[\begin{array}{cc} k_{2} & \\ & k_{2} \end{array}\right]\left\{\begin{array}{l} x^{3}\\ y^{3} \end{array}\right\}=\left\{\begin{array}{l} P_{x}\\ P_{y} \end{array}\right\}+m\mu \omega^{2}\left\{\begin{array}{l} \cos\omega t\\ \sin\omega t \end{array}\right\}+\left\{\begin{array}{l} 0\\ -mg \end{array}\right\},$$

where, *μ* represents eccentricity of the rotor.

With the transformation of $x=\frac{x}{\delta }$, $y=\frac{y}{\delta }$, $\varpi =\frac{\omega }{\omega_{0}}$, τ=ωt, $\frac{c}{m}=2\sigma \omega_{0}$, $\beta =\frac{k_{c}}{k_{1}}$, $\bar{\mu }=\frac{\mu }{\delta }$, the differential equations of motion for the rotor are obtained:

(23) $$\{\begin{array}{c} \ddot{x}+2\sigma \dot{x}+x+\lambda x^{3}+\beta (1-\frac{1}{r})\left[x-fy-\alpha ({\dot{x}}^{2}+{\dot{y}}^{2}{)}^{1/2}\right]=m\varpi \cos\varpi t\\ \ddot{y}+2\sigma \dot{y}+y+\lambda y^{3}+\beta (1-\frac{1}{r})\left[fx+y+\alpha ({\dot{x}}^{2}+{\dot{y}}^{2}{)}^{1/2}\right]=\bar{\mu }\varpi^{2}\sin\varpi t-mg \end{array}.$$

Using the four order Runge-Kutta method to solve Equation (23), we can get the vibration response of the signal in the direction of X and Y. For the simulated signal in this paper, the specific parameters were initialized to: m=2.8 kg, σ=0.4, ω=3, λ=0.45, β=1.65, f=0.1, α=0.3 and $\bar{\mu }=2$.

**Figure 5:**
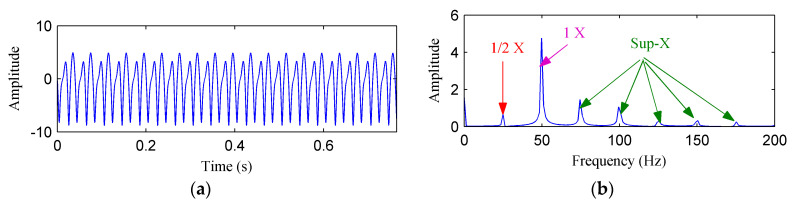
(**a**) The simulated rubbing fault signal; (**b**) its frequency spectrum.

Figure 5a,b shows the waveform and frequency spectrum of the simulated rubbing signal in the Y direction, respectively. As can be seen from Figure 5b, the fundamental frequency, fractional harmonics and high harmonics are all clearly presented.

**Figure 6:**
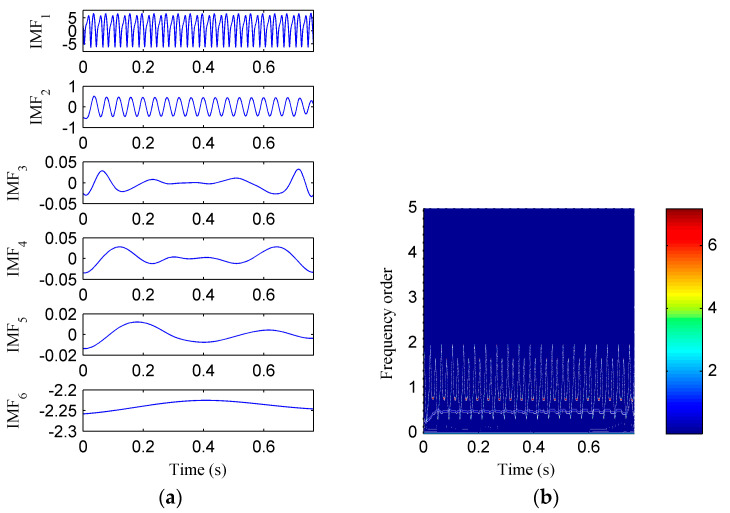
(**a**) The decomposed sub-components using EMD; (**b**) the corresponding HHT time–frequency spectrum.

**Figure 7:**
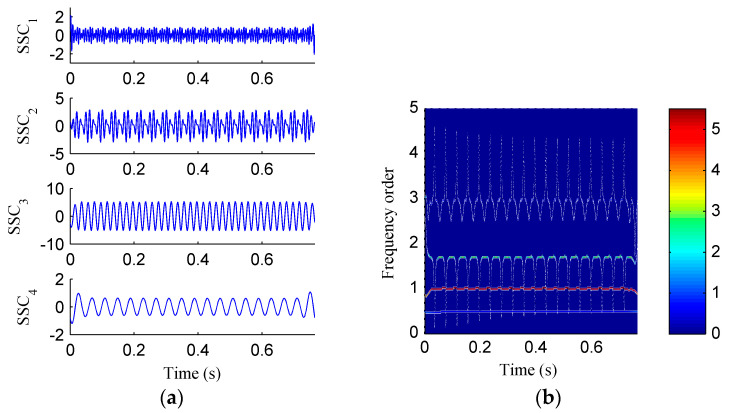
(**a**) The decomposed sub-components using SSD; (**b**) the corresponding SSD-HT time–frequency spectrum.

**Figure 8:**
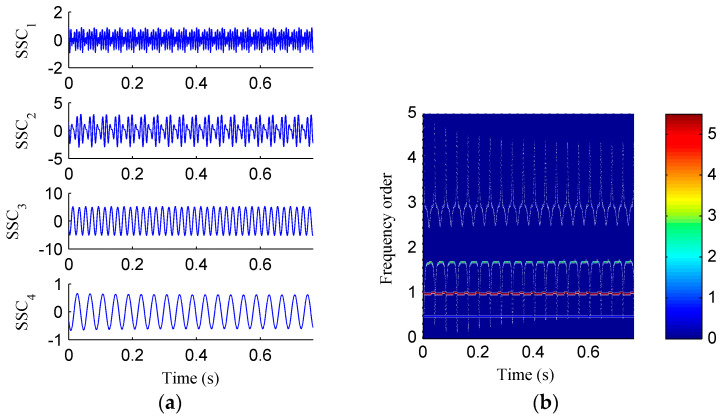
(**a**) The decomposed sub-components using ISSD; (**b**) the corresponding ISSD-HT time–frequency spectrum.

Then, the simulation signal is processed by EMD, SSD and ISSD respectively, and the results are shown in Figure 6, Figure 7 and Figure 8, respectively. Figure 6a displays the decomposed sub-components using EMD and Figure 6b depicts the corresponding HHT TFS, i.e., the EMD-HT TFS. To clearly reveal the relationship between the frequency components exhibited in the TFS and the rotation frequency, the y-axis of the TFS in this paper was described using the frequency order, which is defined as the ratio of the actual frequency to the rotation frequency. Six sub-components were extracted after the EMD decomposition, but some of them are false components without physical meaning. The model aliasing problem appeared on the HHT TFS, which makes the HTT TFS cannot accurately reflect the fault information. As shown in Figure 7a, four sub-components were derived after the SSD decomposition. The corresponding SSD-HT TFS as depicted in Figure 7b reflects the characteristic frequencies of the simulated rubbing fault. Moreover, the “intrawave frequency modulation” phenomenon which is one of the characteristics of rubbing fault as reported in [35] was clearly extracted. However, the end effects can also be found from the SSD-HT TFS. The appearance of the end effect can cause the change of the energy–frequency–time distribution in the TFS to some extent. Consequently, the energy–frequency–time distribution of the TFS will lose its real regularity. To ensure a more accurate measure of the energy distribution characteristics of the TFS by using the CFBEE index, the end effect problem should be solved. Compared with Figure 7a, the sub-components obtained by using ISSD as shown in Figure 8a overcome the end effects. The corresponding ISSD-HT TFS displayed in Figure 8b is also away from the end effects. Hence, the superiority of ISSD method over the other two methods can be demonstrated from the simulated results.

Finally, the CFBEE of the ISSD-HT time–frequency spectrum shown in Figure 8b was calculated as [0.0111, 0.2689, 0.1326 1.1420, 0.0544, 0.3804, 0.7641, 0.0169]. It is visible that the values of the energy entropy of different characteristic frequency bands are different. As the regularity and complexity of the normalized energy sequences of different characteristic frequency bands are different and the entropy index can measure the regularity and complexity of the signal [13,14,16,30], the local information of the energy distribution around the characteristic frequency bands can be measured by the CFBEE indicator. Sometimes, the frequency components in the time–frequency spectrums of different rotor states are similar and difficult to distinguish by naked eyes, the CFBEE indicator can be used as an automatic fault feature extraction method to estimate the differences between different rotor states.

The vibration signal of the rotor collected in the practical operating condition is frequently influenced by external noise [6]. To further verify the effectiveness of the developed method in extracting the fault characteristics from the noisy vibration signals. Additive Gaussian random noise *n*(*t*) was simulated by employing the following equation:

(24) n(t)=0.3randn(1,N),

where *N* represent the length of *n*(*t*).

**Figure 9:**
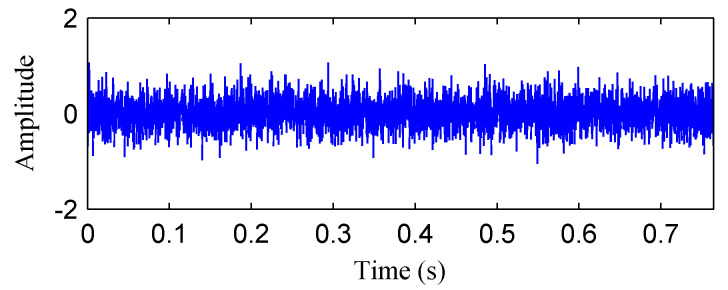
The waveform of the additive noise.

**Figure 10:**
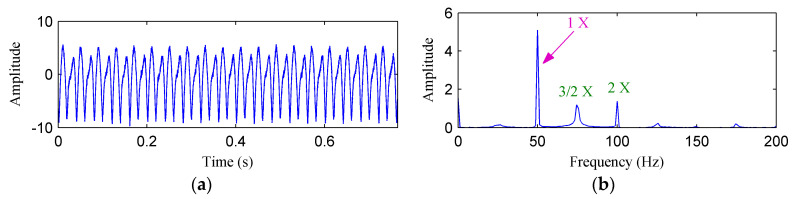
(**a**) The mixed signal of the simulated rubbing fault signal and additive noise; (**b**) its frequency spectrum.

Figure 9 shows the waveform of the additive Gaussian random noise. Then, the obtained noise was added to the simulated rubbing fault signal shown in Figure 5a to generate a mixed signal, which was shown in Figure 10a. Figure 10b describes the FFT spectrum of the mixed signal. Some harmonics of the rotation frequency disappeared compared to Figure 5b due to the influence of noise. It indicates that the adding noise weakened the fault features.

**Figure 11:**
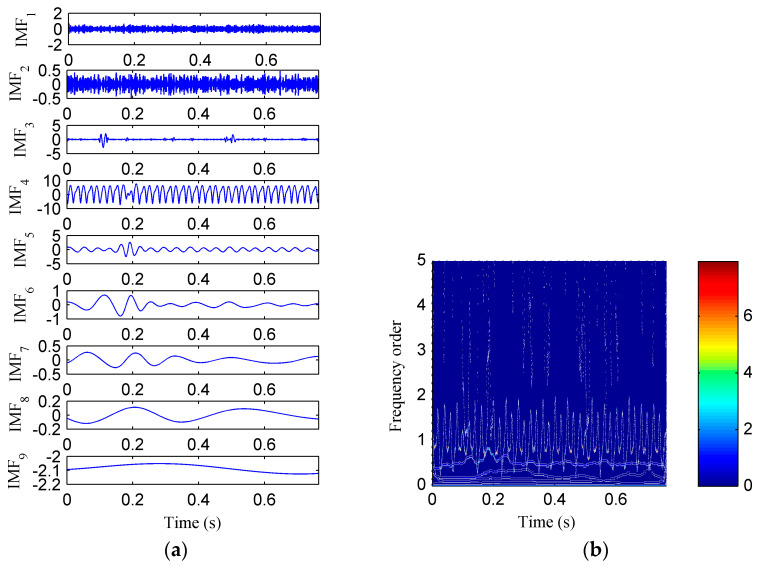
(**a**) The decomposed sub-components of the mixed signal using EMD; (**b**) the corresponding HHT time–frequency spectrum.

**Figure 12:**
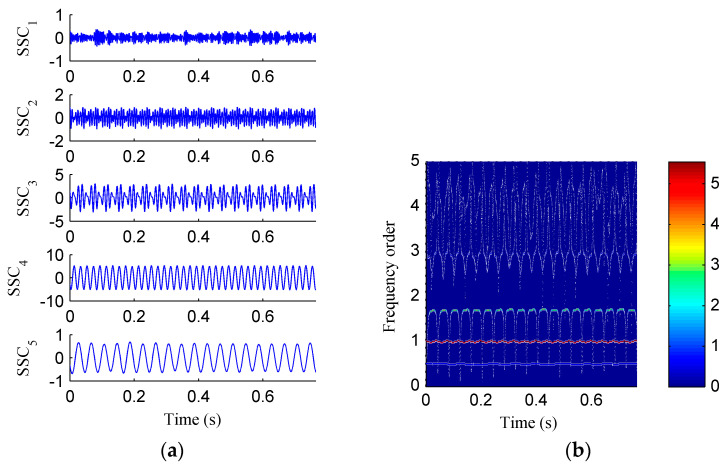
(**a**) The decomposed sub-components of the mixed signal using ISSD; (**b**) the corresponding ISSD-HT time–frequency spectrum.

The mixed signal was analyzed by EMD and ISSD, and the corresponding results can be found from Figure 11 and Figure 12, respectively. Nine sub-components were generated by performing EMD on the mixed signal as Figure 11a shows. That means the additive noise leads to the appearance of more decomposition components without physical meaning. Consequently, the problem of mode aliasing in the corresponding EMD-HT TFS displayed in Figure 11b becomes more serious. As shown in Figure 12a, five sub-components were derived after the ISSD decomposition, the first sub-component represents the noise and the other four components belong to the fault feature components. Figure 12b shows the corresponding ISSD-HT TFS. Despite some high-frequency noise appeared on the ISSD-HT TFS, the fault characteristic frequencies and the “intrawave frequency modulation” characteristics can be clearly identified. It demonstrated that ISSD is more robust to noise compared to EMD.

## 5. Experimental Result and Analysis

### 5.1. Experimental Rig and Data Description

**Figure 13:**
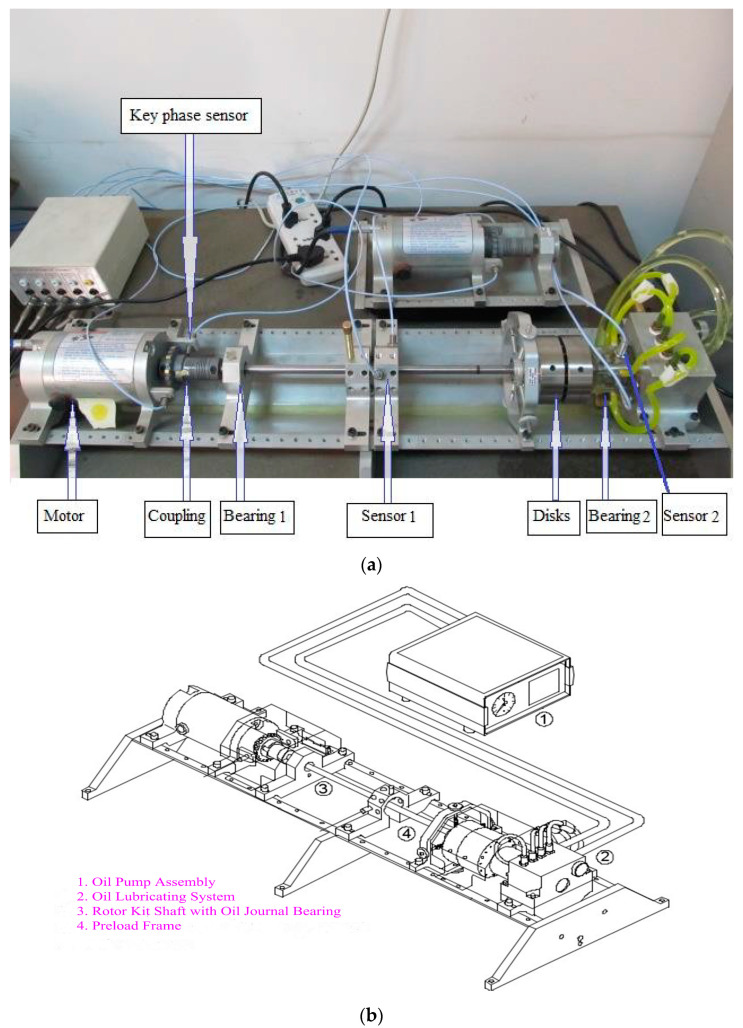
(**a**) Bently RK4 rotor experimental rig; (**b**) its structure diagram.

The experiments were carried out on a Bently RK-4 rotor test bed as displayed in Figure 13a,b shows the structure diagram of the test bed. As can be seen from Figure 13, the test stand is an oil-fluid supporting rotor bearing system, which is mainly built with a motor, a shaft, two discs, several mounting blocks for probe and rub screw, two bearings and a speed regulating device. The test bed can be adopted for simulating some common rotor faults such as rubbing, imbalance and oil film instability failures (see Hu et al. [35] and Wang et al. [36]). During the experiments, a key phase senor was arranged near the rotating shaft to acquire the rotation speed information. Two sets of eddy current sensors which were installed on two mounting blocks were employed to collect the vibration signals of the shaft.

**Figure 14:**
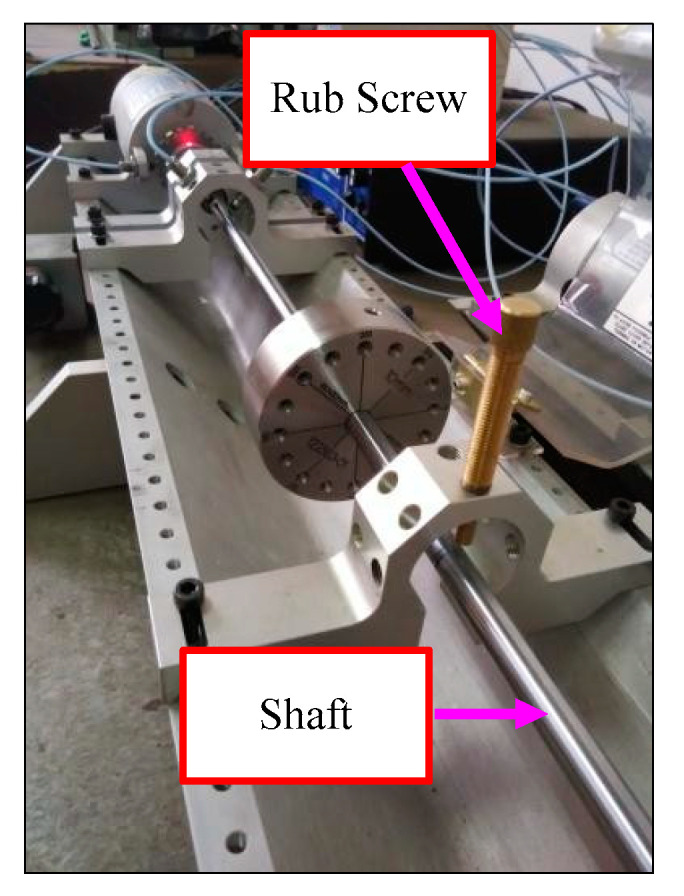
The schematic diagram of rubbing.

To verify the validity of the proposed method, four kinds of rotor fault types, which include normal state, rubbing, oil film whirl and imbalance fault were simulated using the experimental rig. Specifically speaking, a single disk was installed on the shaft for the normal, rubbing and imbalance rotor states simulation. Two bearings were symmetrically arranged on both sides of the disk. The first-order critical speed of the experimental rig is about 1890 rpm. As the working speed of many practical machines, such as the steam turbine, is above the first-order critical speed, the rotation speed of the normal state in this paper was set as 3000 rpm and the test rig was not subjected to any abnormal forces. Since the local rubbing fault is more common than the full rubbing fault, the rotor/stator local rubbing was simulated in the experiment. Figure 14 displays the schematic diagram of rubbing. A rub screw installed on a mounting block was adjusted to touch the shaft to simulate the rub process. The rotation speed for the rubbing sate simulation was kept as 1760 rpm. The rotor imbalance state was simulated by installing counterweight bolts on the disc. The counterweight is 1 g and the rotation speed of the imbalance was set as 1500 rpm. The occurrence of oil film whirl needs to satisfy two conditions: (1) the rotation speed is above a certain speed, which is usually the first-order critical speed; (2) the oil film force is instable. When simulating the oil film whirl state, the following equipment, i.e., oil pump assembly, oil lubricating system, rotor kit shaft with oil journal bearing and preload frame, were required to achieve the instable oil film force. The way this equipment was linked is shown in Figure 13b. Two disks were installed on the shaft for simulating the oil film whirl fault. The rotation speed increased from zero by adjusting the motor and light oil film whirl began to appear at the speed of 1800 rpm. The oil film whirl signal at the speed of 3000 rpm was collected for analysis. The corresponding rotation frequency of the normal, rubbing, oil film whirl and imbalance states can be calculated to 50 Hz, 29.33 Hz, 50 Hz and 25 Hz, respectively. The sampling frequency is 1280 Hz. To evaluate the classification accuracy of the proposed method, the vibration signal of each fault type was dived into 40 samples and the sampling number of each sample is 2048. Among the 40 samples of each rotor state, the former 20 samples were selected as the training sample sets and the other 20 samples were employed as the test sample sets.

### 5.2. The Proposed Method Analysis

**Figure 15:**
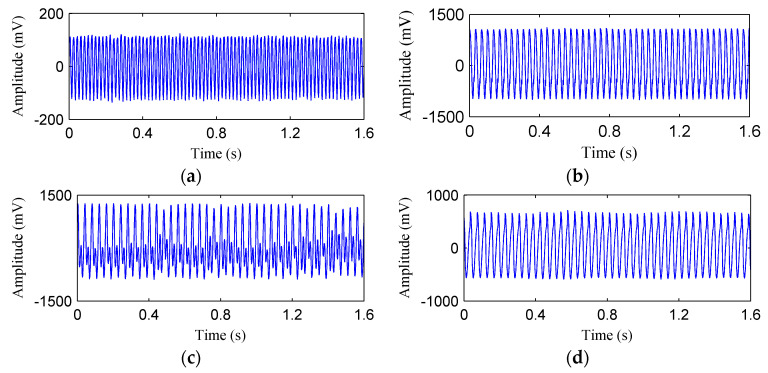
The waveforms of the signal of (**a**) Normal state; (**b**) rubbing state; (**c**) oil film whirl state; (**d**) imbalance state.

**Figure 16:**
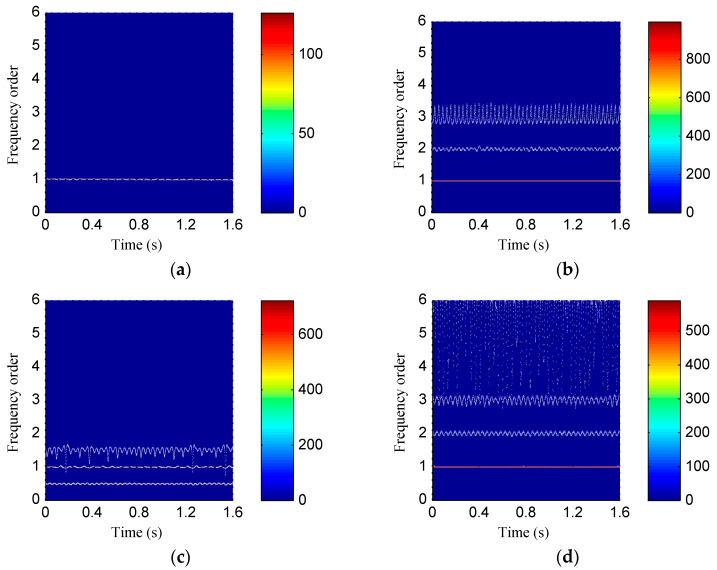
The ISSD-HT time–frequency spectrum of (**a**) Normal state; (**b**) rubbing state; (**c**) oil film whirl state; (**d**) imbalance state.

Figure 15 shows the time waveforms of the rotor vibration signals corresponds to four rotor states. Then both the ISSD and EMD methods were employed to perform the time–frequency analysis on the rotor vibration signals. Figure 16 shows the ISSD-HT TFS of the four rotor states. From Figure 16, we can find that there are obvious differences among the four rotor fault types. For the normal state, only the 1X harmonic of the rotation frequency, i.e., 50 Hz, could be found and its amplitude is relatively small. The ISSD-HT TFS of the rubbing state is composed of the fundamental frequency and its 2X and 3X harmonics, i.e., 29.33 Hz, 58.66 Hz and 87.99Hz. For the oil film whirl state, the fundamental frequency and its 0.5X and 1.5X harmonics, i.e., 50 Hz, 25Hz and 75Hz, can be detected from the ISSD-HT TFS. The main frequency component reflected in the ISSD-HT TFS of the imbalance state include the fundamental frequency (25 Hz), the 2X harmonic frequency (50 Hz), the 3X harmonic frequency (75 Hz) and some other discrete high-frequency components. The ISSD-HT TFS displays the fault characteristics of rotor fault accurately and different fault types can be distinguished apparently. Hence, the energy distribution characteristics of the ISSD-HT TFS can be extracted to distinguish the rotor states.

**Figure 17:**
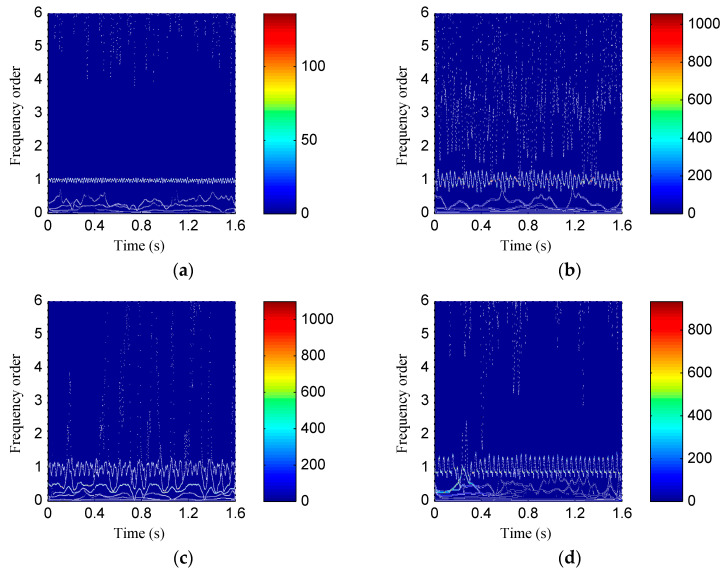
The EMD-HT time–frequency spectrum of (**a**) Normal state; (**b**) rubbing state; (**c**) oil film whirl state; (**d**) imbalance state.

Figure 17 shows the HHT TFS, i.e., the EMD-HT TFS of the four rotor states. From Figure 17, it can be visible that the energy of the TFS of the four rotor states concentrates near the fundamental frequency, and there are no obvious differences among different states. At the same time, the fault characteristic frequencies of rotor faults are hard to be detected from the EMD-HT TFS due to the serious mode problems. The ISSD-HT TFS describes the fault signatures of rotor faults more accurately compared with the EMD-HT TFS.

**Figure 18:**
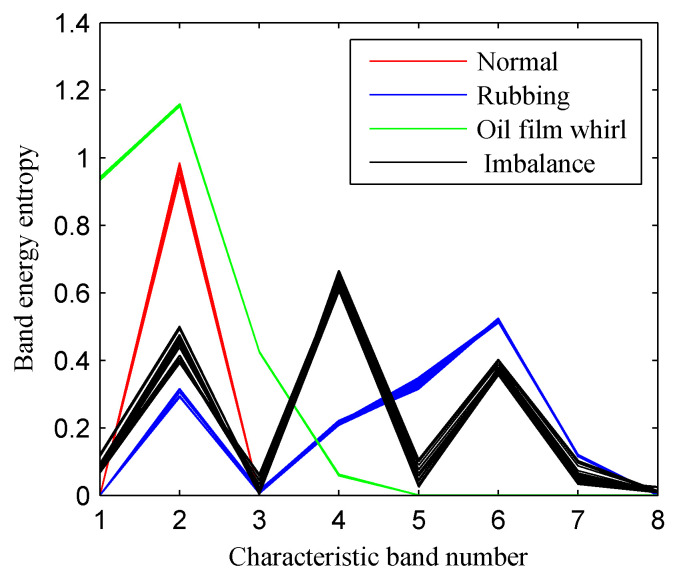
The characteristic frequency band energy entropy of the four rotor states obtained using the proposed method.

**Figure 19:**
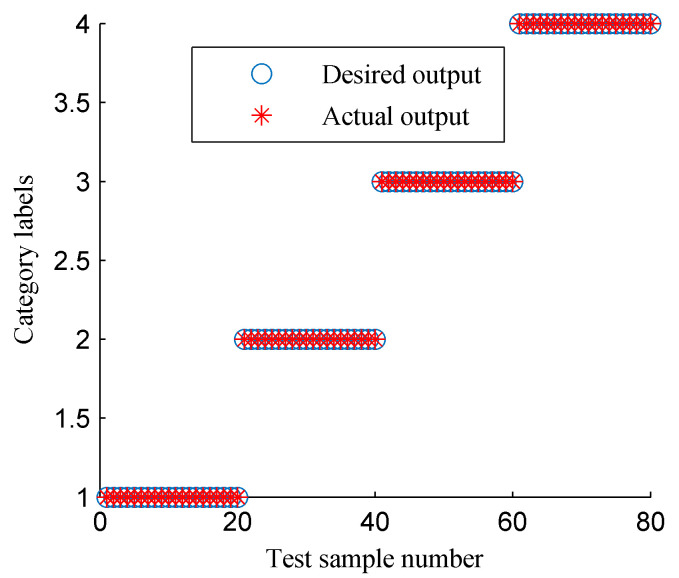
The classification results of SVM using the fault features shown in Figure 18.

Then, the proposed approach was employed to realize the intelligent fault diagnosis of rotor faults. First, the CFBEE of the ISSD-HT TFS of the four simulated states was calculated by using the calculation method introduced above. Figure 18 shows the cure of the CFBEE (each state includes 40 samples). It is obvious that the tendency of feature distribution for a specific rotor state is almost the same, and different rotor states have different trends. Thus, the state of the rotor can be characterized effectively by using the CFBEE index. To diagnose the state of the rotor automatically, SVM was adopted. The calculated CFBEE as the fault feature vectors were input to SVM for training and classifying. The classification results of the test sample sets obtained using SVM is shown in Figure 19. The category label 1, 2, 3 and 4 respect the normal state, rubbing state, oil film whirl state and imbalance state, respectively. The state diagnosis accuracy of the proposed method is 100%. This indicates the proposed approach is very effective for identifying rotor faults.

### 5.3. Comparison with Other Methods

To illustrate the superiority of the proposed methodology, comparative analysis was carried out among the proposed method and three other methods. The first comparison method is the EMD-based CFBEE method. In this method the rotor vibration signal was decomposed by EMD to derive the sub-components and the EMD-HT TFS was used to extract the time–frequency information of different rotor states. Then the CFBEE of the EMD-HT TFS was calculated as the fault features. The Second comparison method is the EMD-based time–frequency entropy (TFE) method which was developed in [17]. The entropy of the whole EMD-HT TFS was calculated in this method. The third comparison method is the ISSD-based TFE method. In this method, the fault signatures were extracted by calculating the TFE of the ISSD-HT TFS.

**Figure 20:**
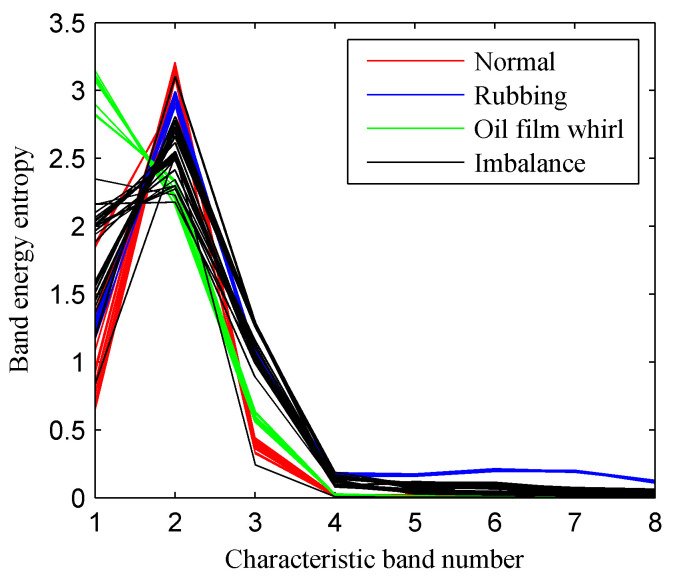
The EMD-based characteristic frequency band energy entropy of the four rotor states.

**Figure 21:**
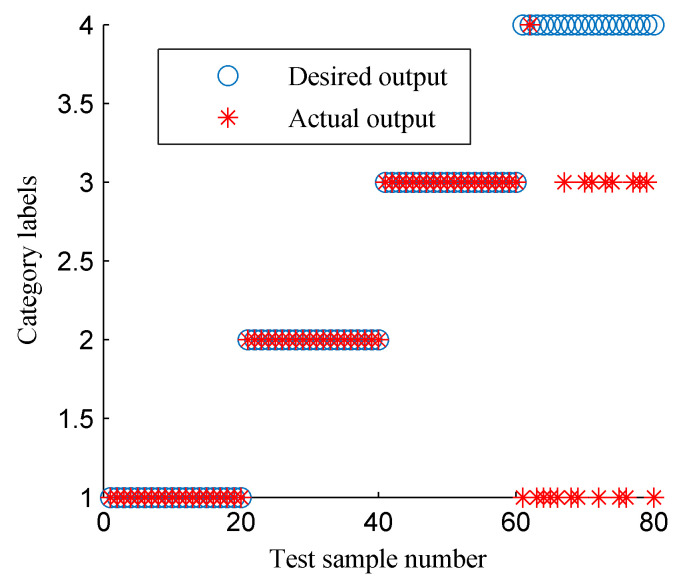
The classification results of SVM using the fault features shown in Figure 20.

Figure 20 displays the curve of the CFBEE of the simulated rotor states (each state includes 40 samples) derived by using the EMD-based CFBEE method. The EMD-based CFBEE curves of the four states show a similar trend of change. Hence, it is difficult to distinguish these four faults by referring to the EMD-based CFBEE. The EMD-based CFBEE was then input to SVM as the feature vector for the automatic classification of rotor sates and the results were given in Figure 21. 19 samples of out of balance state are misadvised, and the classification accuracy is 76.25%, which is lower than the proposed method result. It can be concluded that ISSD is more suitable for processing rotor vibration signals compared to EMD.

**Figure 22:**
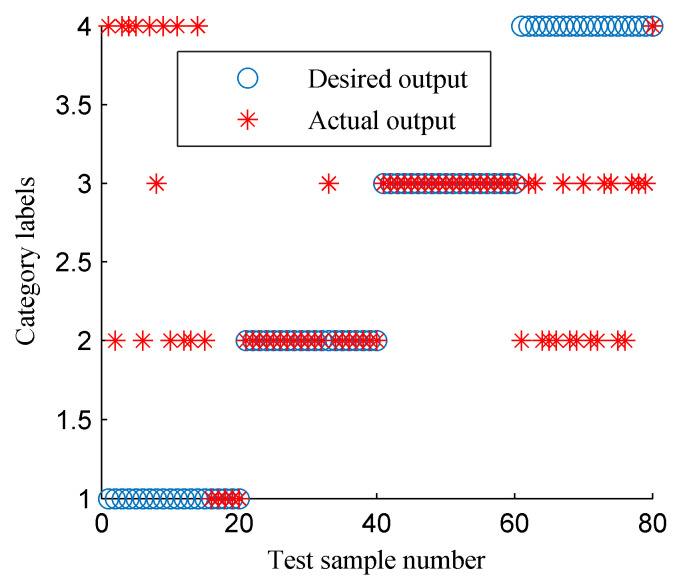
The classification results of SVM using the EMD-based time–frequency entropy.

**Figure 23:**
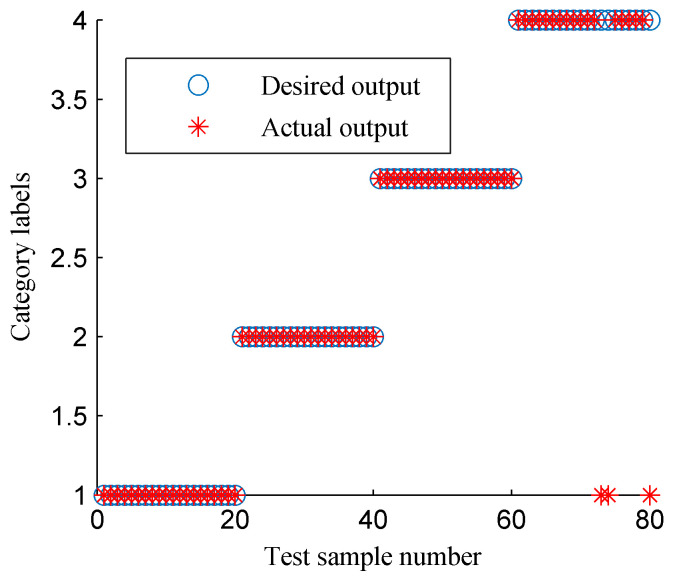
The classification results of SVM using the ISSD-based time–frequency entropy.

Figure 22 and Figure 23 display the classification results of SVM by using the EMD-based TFE and ISSD-based TFE methods, respectively. For the classification results shown in Figure 22, 28 samples of four rotor states are misidentified, so the identification accuracy of the EMD-based TFE method is only 56.25%. Three samples were mistakenly by using the ISSD-based TFE method as Figure 23 shows, therefore the identification accuracy of the ISSD-based TFE method is only 96.25%. The contrast analysis results show that the CFBEE index can more accurately evaluate the characteristic information of the TFS compared the TFE index.

To make a detailed comparison, the classification accuracy for each rotor state corresponding to the proposed method and its three comparison methods was listed in Table 1. The superiority of the proposed method can be clearly demonstrated.

## 6. Conclusions

An improved singular spectrum decomposition method was developed by adopting a novel waveform matching continuation method to improve its end effects. The improved singular spectrum decomposition method was combined with Hilbert transform to perform time–frequency analysis on rotor vibration signal. A novel index called characteristic frequency band energy entropy was introduced to replace the time–frequency entropy to extract the fault feature of the time–frequency spectrum. The state of rotor can be automatically classified by SVM using the fault features of characteristic frequency band energy entropy. By analyzing the results of simulation and experimental signals, we can get the following conclusions:

(1) ISSD can overcome the drawbacks of EMD, such as mode mixing and meaningless components. Moreover, the ISSD method improves the end effects of SSD. Therefore, ISSD is more suitable for extracting the sub-component signals of rotor vibration signals.

(2) The proposed method can identify rotor faults accurately. Compared with EMD-based characteristic frequency band energy entropy method, EMD-based time–frequency entropy method and ISSD-based time–frequency entropy method, the presented method has higher recognition accuracy.

## Figures and Tables

**Table 1:** Identification accuracy of different approaches.

	Normal	Rubbing	Oil Film Whirl	Imbalance	Average Accuracy
The proposed method	100%	100%	100%	100%	100%
The EMD-based CFBEE method	100%	100%	100%	5%	76.25%
The EMD-based TFE method	25%	95%	100%	5%	56.25%
The ISSD-based TFE method	100%	100%	100%	85%	96.25%
